# Correspondence

**Published:** 1866-06

**Authors:** 


### Correspondence.

EDITOR OF DENTAL REGISTER.—As your readers are nearer, or at any rate *dearer* to me than the rest of the profession, will you allow me a little space for a personal matter? A small matter it is, too, and one which I had, for a time, quite forgotten, and which, in some respects, would appear more appropriate in the "Dental Cosmos" than in the "Register" but the dental editor of the "Cosmos," as I understand his editorial and other statements, is so overwhelmed with "the cares of this life," and especially with the heavy responsibility of the entire profession resting on him, that only by a rigid economy of his spare moments, by "working while others sleep," by burning gallons of midnight oil, has he barely time to villify or slander a brother, but not a moment to spare for palliation, correction, or retraction. Oh, no! "life is to short for such encounters, and one should value his *time* and *opportunties* for self-improvement too highly to waste them in that way." So, unless I can get the American Dental Association to let him off with a half dozen speeches on each subject, and to forgo the pleasure of listening, at our next meeting, to his eloquent lecture on the toe-nails, I have no hope of his ever having *time to waste* in doing a simple act of justice in the "Cosmos." I hope, therefore, you will hear me in the "Register."

As you are aware, I prevented the January number of the "Cosmos" from going to the profession without editorial matter. By April I had inaugurated another improvement, in the shape of a PRIVATE CORRESPONDENCE department, which has started off quite racy. Indeed, by the close of the year, I hope to have the "Cosmos" almost as lively as the "Register." Here is an extract from this department; but, as it is *private* correspondence, please don't read it to any one:—

"DEAR McQUILLEN:—Yesterday's note, containing copy of a note from Prof. Watt, is before me, to which I reply, that you have saved me the necessity of responding to Watt, as you reported me as saying just exactly what I meant to say.

Watt did charge me at Chicago with saying that royal jelly changed the sex, which I denied having said or meant. I can not now more than then state just what I did say; but I know what I meant then and now.

I think your stricture correct, just, and necessary. If you understood me to say at Chicago just exactly what I meant to say, I am satisfied that the misapprehension was of the ears of those listeners who now say that I put forth babbling nonsense. If they understood me to say what they now say I said, there is but one conclusion to come to of two, both uncomplimentary to them : 1st, they either did not know I was wrong, or 2nd, they did not care enough for so important a truth as to advocate it against false authority.

As ever

ATKINSON.

" Reported me as saying just exactly what I meant to say." Exactly ! Everybody knows Dr. A. *means* to tell *just exactly* the truth, in science as in other things; but, when he gets confused and blunders, he *doesn't always do it.* " Denied having said or meant." Well, at the social meeting at Prof. Miller's, in less than five minutes after I got there, I called Dr. A.'s attention to his mistake, which was made only a few minutes before adjournment; and I have only to say that Dr. A.'s mode of denial is as *original* as his genius in other things. His first remark was, " Why didn't you pitch into me?" To which I replied, that I thought it was better to call his attention to the matter, that he might correct it himself. For this he thanked me, and added, " I guess I must have waded there." *Now*, I recognize this as a *most emphatic denial*; but I didn't so understand it *then*. You will notice that Dr. A. does not pretend to remember what he did say, and says he didn't remember it even the same evening. But President Spalding, it seems, had a better memory, as his letter to the "Register" illustrates.

The " uncomplimentary " conclusions suggested are so unworthy of Dr. A., that nothing can induce me to hold him responsible for them. They are the inspiration of cerebro-spinal irritation, ("evil angels") and not the *thoughts of William H. Atkinson.* Didn't know? Then who taught us before we met him ? Didn't care ? Then why so promptly (yet so privately) call his attention to the matter as a mistake of a hurried speech? When the "good angels" (healthy nerve forces) get possession of Dr. A. again, he will, instead of these, draw the correct conclusion that, while we noticed his mistake we cared for truth in science, and cared also *for his feelings*. And that is the conclusien Dr. A. *meant* to draw, in the very same sense that he "meant to say" what the "Cosmos" editor has put into his mouth, that is, he intended to be scientific and *truthful* all the time, but partially failed through inadvertence then, and nervous irritability now.

I listen to Dr. A. with the ears of a reporter. I believe I have succeeded better in reporting him than any other non-phonographic reporter in the profession; and he has told me this himself. Dr. Spalding is also an experienced reporter. He heard as I did, and called Dr. A.'s attention to the mistake before they left the hall; and there was no denial then, except to say, in substance, "Why didn't you correct me?" Negative testimony amounts to nothing against positive. It matters not how many didn't hear the remark. Those who did hear it know what it was.

But even if my statement were not true, it does not follow that the editorial contradiction was not coarse, ungentlemanly, and vindictive. Nor does it help the matter that, in May and August preceding, some kind things were said of me, the leading idea being, Dr. W. is a good fellow, *and he likes me.* Joab spoke kindly to his cousin, and then stabbed him under the fifth rib while pretending to kiss him; and the wisest monarch of the world put him to death for it, without a thought of his kind words. And, no doubt, Judas bought the best in market for his Master to eat; but he *betrayed him with a kiss*, and God and man estimate his character accordingly. And Judas never had the impudence to parade his former friendship as an excuse for the betrayal, but had the decency to sneak away and hang himself; and how well it would be for our profession and the world, if those like him would follow his "illustrious example," I leave others to judge.

It may appear to the reader of this that "the game is not worth the candle," but *truth* and *honor* are not yet unmeaning terms in our profession; and that they may ever be held at their true value is the wish of Yours

GEO. WATT.

PROF. J. TAFT.—*Dear Sir.* I saw an article in the "Dental Register" in relation to Dr. E. A. Bogue's improvement on Artificial Palates. Now, this process is not entirely new, as I have made them in a similar way for years. I have been in the practice of inserting Palates for the last fifteen years upon Gold, Silver and Rubber. I have made them upon Gold which have been worn for fifteen years with good satisfaction. I made the back part thin and flexible,, with a downward curve, resting lightly against the muscles of the Pharynx forming a curtain, arched in the center or soft palate, so as to give it the shape of a well-formed mouth and flexible enough, so as not to irritate the muscles in swallowing or speaking. It requires judgement and skill in getting a good impression, and forming and adjusting the Plate. Since the Rubber has been introduced into our practice, I have made much improvement in the manufacture and adjustment of these plates. Now we know that it is not neccessary to fill the nasal cavity, but it is desirable that the plates should be so formed as to give the mouth, or palate and throat, the natural shape. The whole apparatus should be thin, light and flexible, consistent with strength and durability, I have made them of rubber, which accomplishes this desirable object some of which plates have been worn five or six years with the best of satisfaction. It seems that Dr. Bogue's plan is in part, similar to my own in the form of the plate, but in detail much different. The object. as I have already stated is to bring the palate and throat into proper shapes, in order to obtain a perfect articulation, and these plates must be so nicely fitted as to make it tight to prevent the air and sound from passing through the nasal organs, and so flexible as to give full play to the muscles. My practice has been, when the teeth were entirely out and nothing but the alveolar ridge left, to get a good impression of the ridge and back part of the throat as possible, trim the surplus from the top of the impression, make a line at the back part of the alveolar ridge, take the impression of the interior of the ridge, making a line corresponding with the one already taken, get up the plaster models, join them together, then get up a plate of gutta percha and fit it exactly to the mouth, taking the articulation of the teeth in the usual way, adjust the teeth, and bed the whole in the flask (which by the way must be larger and longer than the ordinary ones in use, say three inches in depth and five inches in length. Separate the flasks and remove the Gutta-percha &c., and pack the rubber; the hard around the teeth and alveolar ridge and nearly as far back as the hard palate extends, then commence and blend in the soft rubber until it runs out into all soft rubber on the Sides and back against the Phryngeal Muscles.

I think it preferable to have the plate over the anterior of the ridge of hard rubber, as we avoid the heat, and it lessens the collection of mucus.

Where the teeth are in, the method may be varied according to the circumstances in the case, which I will not occupy your space to explain, unless it is required, if so I should be pleased to comply.

I will here say that these plates may be made entirely of hard rubber, by a peculiar process, making that part of it hard where required, and the remainder soft.

SAM. LAWRENCE
Lowell, Mass.

